# Entre o desconhecido e o emergente: mapeando os vírus Oropouche e Mayaro no Brasil

**DOI:** 10.1590/0102-311XPT067525

**Published:** 2026-03-02

**Authors:** Gabriele Nascimento de Oliveira, Léo Shigueki Sato, Antonio Henrique Rosas Novaes, Natan Nascimento de Oliveira, Luciana Dias Ghiraldi Lopes, Dennis Armando Bertolini

**Affiliations:** 1 Universidade Estadual de Maringá, Maringá, Brasil.

**Keywords:** Arboviroses, Análise Espacial, Infecção por Vírus Oropouche, Infecção por Virus Mayaro, Arbovirus Infections, Spatial Analysis, Oropouche Virus Infection, Mayaro Virus Infection, Arbovirosis, Análisis Espacial, Infección por Virus Oropouche, Infección por Virus Mayaro

## Abstract

As arboviroses são doenças infecciosas causadas por vírus transmitidos por artrópodes. Antes da introdução dos vírus Chikungunya e Zika no Brasil, o vírus Oropouche (OROV) era o segundo arbovírus mais prevalente em humanos, atrás apenas do vírus Dengue. Atualmente, os vírus Mayaro (MAYV) e OROV destacam-se como agentes importantes, principalmente na Região Amazônica. Apesar do seu grande impacto, os casos de OROV e MAYV seguem negligenciados e subnotificados. Este estudo teve como objetivo analisar a distribuição geoespacial dos casos de OROV e MAYV para auxiliar na implementação de medidas de controle. Trata-se de um estudo transversal baseado em dados secundários obtidos via Fala.BR, a partir do Gerenciador de Ambiente Laboratorial e do Instituto Brasileiro de Geografia e Estatística sobre febre do Mayaro e febre do Oropouche, incluindo registros laboratoriais e informações populacionais para análise epidemiológica e espacial. Foram analisados 16.571 casos de febre do Oropouche e 379 casos de febre do Mayaro. Os *clusters* espaciais revelam, para a febre do Oropouche, aglomerados de alta incidência (alto-alto) na Região Norte e no Espírito Santo. Já a febre do Mayaro apresenta *clusters* alto-alto no Norte e pontos no Centro-oeste. A sobreposição entre o desmatamento da Amazônia e a incidência dos agravos sugere relação espacial. As áreas de maior incidência coincidem com regiões intensamente desmatadas. O crescimento de casos de febre do Mayaro e febre do Oropouche pode estar ligado à descentralização do diagnóstico e à busca ativa em amostras negativas. Os casos se concentram no Norte, onde fatores ambientais e sociais favorecem a transmissão, especialmente em populações vulneráveis. Há indícios de relação da incidência com o desmatamento. A febre do Mayaro também apresenta aparente associação com a degradação ambiental.

## Abstract (en)

Arboviruses are infectious diseases caused by arthropod-transmitted viruses. Before the introduction of the Chikungunya and Zika viruses in Brazil, the Oropouche virus (OROV) was the second most prevalent arbovirus in humans, behind only Dengue. Currently, the Mayaro virus (MAYV) and OROV stand out as important viral agents, especially in the Amazon Region. Despite the considerable impact, cases of OROV and MAYV remain neglected and underreported. The aim of the present study was to analyze the geospatial distribution of cases of OROV and MAYV to assist in the implementation of control measures. For such, a cross-sectional study was conducted using secondary data obtained via Fala.BR from the Laboratory Environment Manager and the Brazilian Institute of Geography and Statistics on Mayaro fever and Oropouche fever, including laboratory records and population-based information for epidemiological and spatial analysis. A total of 16,571 cases of OROV and 379 cases of MAYV were analyzed. The spatial analysis revealed clusters of the high incidence (high-high) of OROV in the North Region and the state of Espírito Santo. High-high clusters of MAYV were found in the North Region and points in the Central-West. The overlap between deforestation in the Amazon and the incidence of cases suggests a spatial relationship. The areas with the highest incidence coincide with intensely deforested regions. The increase in cases of MAYV and OROV may be linked to the decentralization of the diagnosis and active search in negative samples. The cases are concentrated in the North, where environmental and social factors favor transmission, especially in vulnerable populations. There is evidence of a relationship between incidence and deforestation. MAYV also has an apparent association with environmental degradation.

## Abstract (es)

Los arbovirus son enfermedades infecciosas causadas por virus transmitidos por artrópodos. Antes de la introducción de los virus Chikungunya y Zika en Brasil, el virus Oropouche (OROV) era el segundo arbovirus más prevalente en humanos, solo detrás del virus Dengue. Actualmente, el virus Mayaro (MAYV) y el OROV destacan como agentes importantes, principalmente en la Región Amazónica. A pesar de su gran impacto, los casos de OROV y MAYV siguen siendo desatendidos y subnotificados. Este estudio tuvo como objetivo analizar la distribución geoespacial de los casos de OROV y MAYV para ayudar en la implementación de medidas de control. Se trata de un estudio transversal basado en datos secundarios obtenidos de Fala.BR por medio del Gestor de Ambiente de Laboratorio y del Instituto Brasileño de Geografía y Estadística sobre la fiebre de Mayaro y la fiebre de Oropouche, incluyendo registros de laboratorio e información poblacional para análisis epidemiológico y espacial. Se analizaron 16.571 casos de fiebre de Oropouche y 379 casos de fiebre de Mayaro. Los clústers espaciales revelan, para la fiebre de Oropouche, conglomerados de alta incidencia (alto-alto) en la región Norte y en Espírito Santo. A su vez, la fiebre de Mayaro presenta clústers alto-alto en el Norte y puntos en el Centro-Oeste. La superposición entre la deforestación en la Amazonía y la incidencia de las enfermedades sugiere una relación espacial. Las zonas con mayor incidencia coinciden con regiones que han sido intensamente deforestadas. El aumento de casos de fiebre de Mayaro y fiebre de Oropouche puede estar relacionado con la descentralización del diagnóstico y la búsqueda activa de casos en muestras negativas. Los casos se concentran en la región Norte, donde factores ambientales y sociales favorecen la transmisión, especialmente entre poblaciones vulnerables. Existen evidencias de una relación entre la incidencia de estos eventos y la deforestación. La fiebre de Mayaro también presenta una aparente asociación con la degradación ambiental.

## Introdução

Os arbovírus (vírus transmitidos por artrópodes) representam um grande grupo de vírus que são transmitidos aos humanos pela picada de artrópodes infectados. Esses vírus mantêm-se na natureza em ciclos zoonóticos e são importantes causadores de doenças infecciosas ^1^. Em países tropicais, as arboviroses podem ocorrer de forma endêmica ou epidêmica, emergir em grandes surtos e se tornar problemas globais de saúde pública ^2^. Em 2024, o Brasil passou por uma epidemia de dengue sem precedentes históricos, superando o pior cenário predito por modelos epidemiológicos e a emergência/reemergência do vírus Oropouche (OROV) e do vírus Mayaro (MAYV) ^3^.

O OROV pertence à família *Peribunyaviridae*, do gênero *Orthobunyavirus*, da espécie *Orthobunyavirus oropoucheense*, e é conhecido por causar a febre do Oropouche, caracterizada pela manifestação de sintomas inespecíficos como: cefaleia intensa, dores musculares e articulares, além de náusea e diarreia ^4^. O vírus é envelopado esférico, possuindo três segmentos de RNA de fita simples de sentido negativo ^5^. O OROV é transmitido majoritariamente através da picada de insetos vetores hematófagos, como o *Culicoides paraensis* e o *Culex quinquefasciatus*, sendo o primeiro considerado o principal vetor nas Américas ^6^ ^,^ ^7^.

O MAYV é um arbovírus envelopado, composto por RNA de fita simples de senso positivo, pertencente à família *Togaviridae* e ao gênero *Alphavirus*, conhecido principalmente por também englobar o vírus Chikungunya (CHIKV) ^8^. A fisiopatologia da febre do Mayaro ainda é pouco conhecida, causando uma doença febril aguda, caracterizada por cefaleia e artralgia intensa ^9^. Os primeiros estudos com mosquitos coletados na natureza no Brasil identificaram o *Haemagogus (Haemagogus) janthinomys* como o principal vetor do MAYV ^10^. Posteriormente, estudos experimentais em outros países demonstraram que o *Aedes aegypti* e o *Aedes albopictus* também apresentam competência vetorial para o vírus ^11^.

Durante o período da epidemia de dengue em 2024, a reemergência do OROV causou preocupação ^12^. No Brasil, antes da introdução dos CHIKV e vírus Zika (ZIKV), o OROV era o segundo arbovírus mais prevalente em humanos, sendo superado apenas pelo vírus Dengue (DENV), principalmente em estados da Região Amazônica ^8^. Fatores como mudança climática, desmatamento e urbanização não planejada têm facilitado a disseminação em estados não amazônicos ^13^.

Apesar do seu grande impacto na América do Sul, especialmente na Região Amazônica, os casos de OROV e MAYV continuam sendo negligenciados e subnotificados ^4^, isto aliado ao fato das epidemias de dengue dificultarem a detecção devido à semelhança clínica entre estas e outras doenças infecciosas que ocorrem no Brasil. Diante desse cenário, torna-se fundamental compreender a distribuição dos casos confirmados, de modo a fornecer subsídios para ações de vigilância e controle ^14^. Desta forma, este estudo teve como objetivo analisar a distribuição geoespacial dos casos de OROV e MAYV, no período de 1º de janeiro de 2015 a 12 de janeiro de 2025, de modo a subsidiar a implementação de medidas de controle voltadas principalmente ao monitoramento e manejo dos vetores, bem como à prevenção de novos casos.

## Metodologia

### Desenho do estudo

Trata-se de um estudo transversal realizado no Brasil, baseado em dados secundários sobre febre do Mayaro e febre do Oropouche, incluindo registros laboratoriais e informações populacionais para análise epidemiológica e espacial.

### Participantes

Foram incluídos todos os casos de febre do Mayaro e febre do Oropouche confirmados por biologia molecular (RT-PCR) ou sorologia IgM ELISA, conforme registros do Gerenciador de Ambiente Laboratorial (GAL). Os dados foram solicitados à Secretaria de Vigilância à Saúde do Ministério da Saúde (SVS/MS) pelo Fala.BR (https://falabr.cgu.gov.br/web/home; protocolo nº 25072.065860/2024-51), que, após aprovação, enviou os dados de forma anonimizada e previamente processada, incluindo informações demográficas e clínicas estratificadas por Unidade Federativa e município de residência. Como os dados utilizados são secundários e em conformidade com as *Resoluções nº 466/2012* e *nº 560/16* do Conselho Nacional de Saúde, a apreciação pelo Comitê Permanente de Ética em Pesquisa (COPEP) foi dispensada. No entanto, os pesquisadores seguiram rigorosamente os mesmos princípios éticos estabelecidos.

### Fontes de dados

Os dados foram obtidos através da SVS/MS, via Fala.BR, incluindo todos os anos disponíveis e todos os municípios brasileiros, a partir do GAL e do Instituto Brasileiro de Geografia e Estatística (IBGE; https://www.ibge.gov.br/), garantindo uma base padronizada para análise. O período analisado compreendeu os registros de 1º de janeiro de 2015 a 12 de janeiro de 2025, correspondente ao intervalo completo de dados disponibilizado pela SVS mediante nossa solicitação para o total de casos de febre do Oropouche e febre do Mayaro. O processamento foi realizado no software R (http://www.r-project.org), com procedimentos de limpeza, organização e tabulação em bancos de dados separados para cada agravo.

### Variáveis

As análises consideraram dados laboratoriais (tipo de exame e resultado), informações demográficas (idade, sexo e local de residência) e clínicas, além de dados populacionais para o cálculo de indicadores epidemiológicos. Também foram incorporadas informações sobre o desmatamento na Amazônia e na Amazônia Legal, obtidas do Instituto Nacional de Pesquisas Espaciais (INPE; https://terrabrasilis.dpi.inpe.br/) - Projeto de Monitoramento do Desmatamento na Amazônia Legal por Satélite (PRODES). As variáveis numéricas incluíram indicadores epidemiológicos calculados com base nos dados populacionais do IBGE e nos registros laboratoriais do GAL.

### Viés

Os possíveis vieses incluem subnotificação e falta de um sistema estruturado de vigilância para as doenças estudadas. Para minimizar esses fatores, foram considerados apenas casos confirmados por exames laboratoriais.

### Métodos estatísticos

Foi realizada uma análise descritiva das variáveis disponíveis. Para a análise espacial, utilizou-se o Índice de Moran Global para avaliar a autocorrelação espacial global e o *Local Indicators of Spatial Association* (LISA; Indicadores Locais de Associação Espacial) para identificar padrões locais de agrupamento e dispersão. Ainda, foi realizada uma análise de Moran Bivariado, com estimação de LISA Bivariado, das taxas de febre do Mayaro e febre do Oropouche com a porcentagem de área desmatada. O cálculo da área desmatada foi feito a partir da razão entre os quilômetros quadrados de área desmatada e os quilômetros quadrados da área do município, multiplicado por 100. Os dados de desmatamento por município foram acessados através da plataforma MapBiomas (https://brasil.mapbiomas.org/). Os cálculos foram realizados nos softwares ArcGIS Pro, versão 3.5.0 (http://www.esri.com/software/arcgis/index.html), e GeoDa, versão 1.18 (https://spatial.uchicago.edu/geoda), gerando mapas e gráficos para visualização dos padrões espaciais.

## Resultados

Foram analisados 16.571 casos de febre do Oropouche e 379 casos de febre do Mayaro, considerando variáveis demográficas, temporais e laboratoriais.

**Figura 1:**
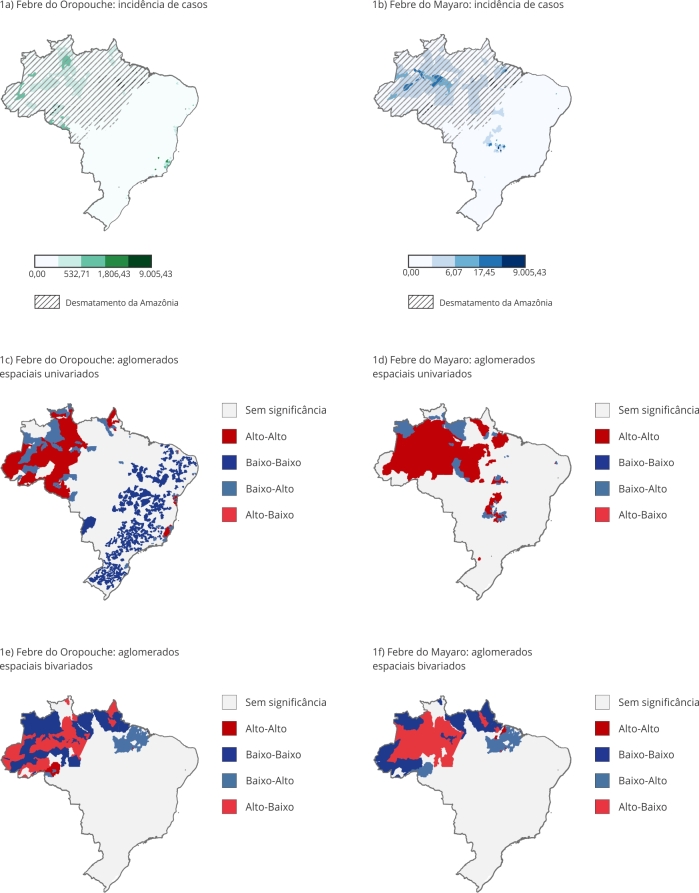
*Clusters* espaciais de casos de febre do Mayaro e de febre do Oropouche no Brasil entre 2015 e 2025.

Na Tabela 1, é possível ver a distribuição das características sociodemográficas e laboratoriais dos casos confirmados. Tanto para febre do Oropouche quanto para febre do Mayaro, há uma distribuição semelhante entre homens e mulheres. A maioria dos casos de febre do Oropouche ocorreu em 2024 (87%), enquanto a febre do Mayaro apresentou o maior número de casos em 2015, com 20% do total do período estudado. A biologia molecular foi o principal método diagnóstico para febre do Oropouche (94%), enquanto a sorologia IgM foi mais utilizada para febre do Mayaro (62%). A média de idade dos pacientes foi semelhante entre as infecções. Em relação à distribuição geográfica, os casos de febre do Oropouche, apresentados na Figura 1a, foram mais frequentes no Espírito Santo e Amazonas, enquanto os de febre do Mayaro, apresentados na Figura 1b, predominou nos estados do Amazonas, Goiás e Pará.

**Tabela 1:** Características epidemiológicas das infecções por febre do Oropouche e febre do Mayaro no Brasil de 2015 a 2025.

Características	Febre do Oropouche (n = 16.571)	Febre do Mayaro (n = 379)
	n (%)	n (%)
Sexo		
Feminino	7.934 (48,01)	200 (53,76)
Masculino	8.630 (51,95)	179 (46,24)
Ignorado	7 (0,04)	0 (0,00)
Ano		
2015	6 (< 0,1)	74 (20,0)
2016	15 (< 0,1)	6 (1,6)
2017	4 (< 0,1)	11 (2,9)
2018	99 (0,6)	13 (3,4)
2019	36 (0,2)	8 (2,1)
2020	13 (< 0,1)	2 (0,5)
2021	94 (0,6)	16 (4,2)
2022	34 (0,2)	11 (2,9)
2023	948 (5,7)	63 (17,0)
2024	14.429 (87,0)	175 (46,0)
2025	893 (5,4)	0 (0,0)
Tipo de exame		
Biologia molecular	15.537 (93,8)	143 (38,0)
Sorologia IgM	1.034 (6,2)	236 (62,0)
Idade do paciente [média (DP)] *	38 (18)	37 (17)
UF de residência		
Acre	548 (3,3)	2 (0,5)
Alagoas	123 (0,7)	0 (0,0)
Amapá	218 (1,3)	6 (1,6)
Amazonas	3.660 (22,0)	140 (37,0)
Bahia	1.095 (6,6)	0 (0,0)
Ceará	263 (1,6)	0 (0,0)
Distrito Federal	1 (< 0,1)	3 (0,8)
Espírito Santo	6.677 (40,0)	0 (0,0)
Goiás	11 (< 0,1)	90 (24)
Maranhão	65 (0,4)	1 (0,3)
Mato Grosso	52 (0,3)	2 (0,5)
Mato Grosso do Sul	9 (< 0,1)	0 (0,0)
Minas Gerais	313 (1,9)	0 (0,0)
Pará	584 (3,5)	113 (30,0)
Paraíba	15 (< 0,1)	1 (0,3)
Paraná	21 (0,1)	7 (1,8)
Pernambuco	232 (1,4)	2 (0,5)
Piauí	48 (0,3)	0 (0,0)
Rio de Janeiro	163 (1,0)	0 (0,0)
Rio Grande do Norte	13 (< 0,1)	1 (0,3)
Rio Grande do Sul	4 (< 0,1)	0 (0,0)
Rondônia	1.735 (10,0)	2 (0,5)
Roraima	466 (2,8)	1 (0,3)
Santa Catarina	182 (1,1)	0 (0,0)
São Paulo	23 (0,1)	0 (0,0)
Sergipe	33 (0,2)	0 (0,0)
Tocantins	17 (0,1)	8 (2,1)

DP: desvio padrão; UF: Unidade Federativa.

* Ignorados: febre do Oropouche - 19; febre do Mayaro - 1.

A análise de *clusters* espaciais (Figuras 1c e 1d) revela padrões distintos para cada doença. No caso da febre do Oropouche, observa-se a presença de aglomerados de alta incidência (alto-alto) predominantemente na Região Norte, com destaque para o Amazonas, Acre e Rondônia (AMACRO), além de um *cluster*, tomando grande parte do Espírito Santo. Os *clusters* de baixa incidência (baixo-baixo) se apresentam dispersos por todo o território nacional, exceto pela Região Norte, onde predominaram os *clusters* alto-alto. Já a febre do Mayaro apresenta *clusters* alto-alto concentrados no Norte, especialmente no Pará, Amazonas e Roraima, com alguns pontos no Centro-oeste.

A análise bivariada entre os dados de desmatamento da Amazônia e a incidência dos agravos (Figuras 1e e 1f) indica relação negativa entre as variáveis, com valores de Moran para febre do Oropouche e febre do Mayaro de -0,153 (p = 0,01) e -0,173 (p < 0,001), respectivamente. Para a febre do Oropouche, áreas de maior incidência antagonizam com regiões intensamente desmatadas no Norte. O mesmo padrão é observado para a febre do Mayaro, onde a maior incidência está inversamente alinhada com áreas críticas de desmatamento.

## Discussão

O aumento significativo do número de casos de febre do Mayaro e de febre do Oropouche trouxe uma nova onda de preocupação em relação à emergência e reemergência de outros arbovírus em um cenário global ^3^. Essa abordagem de análise epidemiológica e espacial é essencial para compreender a distribuição e a situação dessas doenças no Brasil, permitindo uma avaliação mais detalhada de seus padrões geográficos. Esse aumento nos últimos dois anos, como nossos resultados demonstram, pode ser resultado da descentralização do diagnóstico biomolecular destes agravos, aliado à adoção de estratégias como a busca ativa em amostras negativas para dengue, chikungunya e zika em laboratórios sentinela ^12^.

Neste estudo, observou-se uma leve predominância de mulheres entre os casos confirmados de MAYV, em consonância com achados prévios no Brasil ^15^ ^,^ ^16^. A distribuição entre os sexos para febre do Oropouche foi semelhante, sugerindo exposição comparável entre homens e mulheres. Quanto à idade, a média dos pacientes foi próxima entre os agravos, 38 anos para febre do Oropouche e 37 anos para febre do Mayaro, indicando que adultos jovens são os mais frequentemente afetados, possivelmente devido a padrões de exposição relacionados a atividades ocupacionais ou mobilidade em áreas de risco ^17^. Observou-se também diferenças na confirmação diagnóstica: a maioria dos casos de febre do Oropouche foi identificada por biologia molecular (93,8%), enquanto em febre do Mayaro predominou a sorologia IgM (62%), refletindo tanto a disponibilidade de recursos quanto a sensibilidade dos métodos, o que pode impactar a detecção e distribuição dos casos observados ^15^.

Nossos dados apontam para uma concentração de casos em estados da Região Norte. As características ambientais e sociais das diferentes regiões influenciam diretamente na distribuição e cocirculação de arboviroses. Na Amazônia Legal, a presença de vetores e reservatórios em áreas florestais favorece a endemicidade da febre do Mayaro e da febre do Oropouche ^18^, com maior ocorrência em populações ribeirinhas, rurais e indígenas ^4^. Essas comunidades, muitas vezes isoladas geograficamente, enfrentam desafios no acesso a serviços de saúde, diagnóstico e tratamento, o que pode contribuir para a subnotificação e a maior vulnerabilidade à infecção. Além disso, barreiras linguísticas e culturais podem dificultar a implementação de estratégias de prevenção e controle nessas populações ^5^.

Os resultados demonstram uma relação inversa entre os processos de desmatamento na Região Amazônica e a incidência de casos de febre do Oropouche e de febre do Mayaro. Essa relação é corroborada pela literatura, ainda que nem sempre de forma diretamente inversa, ampliando a discussão para aspectos ligados às mudanças climáticas e às alterações no comportamento dos vetores. A relação entre a incidência dos agravos e o desmatamento nas regiões da Amazônia e Amazônia Legal sugere que a perda de cobertura florestal, a urbanização desorganizada e alterações climáticas podem alterar a dinâmica de transmissão, ao modificar habitats de vetores e ampliar a interação entre humanos e reservatórios naturais ^19^.

Atividades que dependem da ampliação de fronteiras através do desmatamento, como a expansão do agronegócio e a construção da BR-319, são evidenciadas principalmente na região AMACRO, a fronteira de desmatamento responsável por grande parte da perda florestal nos estados do Amazonas, Rondônia e Acre entre 2017 e 2021. Esses fatores podem explicar o aumento de casos de febre do Oropouche nos estados do Acre, Amazonas, Rondônia e Roraima, com mais de 6.200 confirmados até março de 2024 ^4^. De maneira semelhante, a febre do Mayaro também tem mostrado uma relação com a degradação ambiental, com a previsão de alta adequação ambiental em várias regiões do Brasil, incluindo áreas da Amazônia ^20^. A expansão agrícola, o desmatamento e o crescimento urbano favorecem a transmissão de ambos os vírus, aumentando o risco de *spillover* viral e de epidemias em áreas recém-colonizadas ^21^.

Por se tratar de dados obtidos através do sistema Fala.BR e devido à falta de um sistema estruturado para os agravos de febre do Mayaro e febre do Oropouche, não foi possível acessar todas as informações contidas nas fichas de notificação, como sinais clínicos, doenças pré-existentes, raça/cor, escolaridade, residência em áreas urbanizadas ou rurais e de ocupação, o que permitiria uma análise mais robusta. A ausência de informações detalhadas sobre o número e a disponibilidade de testes diagnósticos por região limita a interpretação da distribuição observada dos casos. A presença de um *cluster* no Espírito Santo reflete a emergência recente de febre do Oropouche no estado ^22^ e ilustra a capacidade do vírus de se estabelecer fora de seu habitat tradicional na Amazônia. Em áreas não amazônicas, a transmissão de OROV tem sido associada a características rurais e agrícolas que favorecem a reprodução do vetor.

Devido à similaridade dos sintomas iniciais entre as arboviroses em cocirculação, o diagnóstico laboratorial é essencial para a confirmação e a diferenciação dos casos ^3^. Nesse sentido, os exames moleculares representam um avanço imprescindível ^12^, visto que o diagnóstico sorológico pode apresentar falsos positivos devido à reação cruzada entre arbovírus de um mesmo grupo ^23^. Metodologias de sequenciamento genético e de análises filogenéticas podem auxiliar no entendimento da situação que ocorrem no Espírito Santo ou em outros estados, uma vez que a identificação do genótipo de OROV poderia estabelecer a rede de transmissão do vírus e contribuir para tomada de decisões pelo poder público. Esses dados seriam utilizados para melhor entender a epidemiologia molecular e o diagnóstico das infecções pelo OROV ^24^.

## Conclusão

Análises geoespaciais são importantes pois ilustram como ocorre a distribuição de casos das arboviroses e permitem conclusões sobre a dinâmica de distribuição da doença ou, ainda, permitem uma antecipação de estratégias para locais que ainda não foram atingidos. Neste artigo, foi possível evidenciar a distribuição de casos de febre do Oropouche e febre do Mayaro, principalmente na Região Norte, na região da Amazônia, com um aumento significativo no número de casos no ano de 2024, sugerindo maior circulação dos vírus.

Esses achados reforçam a necessidade da vigilância laboratorial e epidemiológica, principalmente em regiões endêmicas onde há a cocirculação de vários arbovírus. As semelhanças clínicas entre essas arboviroses e outras doenças endêmicas dificultam o diagnóstico podendo levar à subnotificação.

Diante do impacto crescente dessas enfermidades, a implantação de políticas públicas de monitoramento contínuo e a integração de dados epidemiológicos, ambientais e climáticos se faz de suma importância para uma maior compreensão dessas doenças, bem como o planejamento de estratégias de prevenção.

## Data Availability

As fontes de informação utilizadas no estudo estão indicadas no corpo do artigo.

## References

[1] Pang M, Sun X, He T, Yang H, Chen J (2025). Clinical manifestation of arboviruses in paediatrics. Rev Med Virol.

[2] Mourão MPG, Bastos MS, Figueiredo RP, Gimaque JBL, Galusso ES, Kramer VM (2012). Mayaro fever in the city of Manaus, Brazil, 2007-2008. Vector Borne Zoonotic Dis.

[3] Ministério da Saúde (2023). Monitoramento das arboviroses e balanço de encerramento do Comitê de Operações de Emergência (COE) Dengue e outras Arboviroses 2024.

[4] Naveca FG, Almeida TAP, Souza V, Nascimento V, Silva D, Nascimento F (2024). Human outbreaks of a novel reassortant Oropouche virus in the Brazilian Amazon region. Nat Med.

[5] Zhang Y, Liu X, Wu Z, Feng S, Lu K, Zhu W (2024). Oropouche virus a neglected global arboviral threat. Virus Res.

[6] Mendonça SF, Rocha MN, Ferreira FV, Leite THJF, Amadou SCG, Sucupira PHF (2021). Evaluation of Aedes aegypti, Aedes albopictus, and Culex quinquefasciatus mosquitoes competence to Oropouche virus infection. Viruses.

[7] Coordenação-Geral de Vigilância de Arboviroses. Departamento de Doenças Transmissíveis. Secretaria de Vigilância em Saúde e Ambiente. Ministério da Saúde (2024). Nota Técnica nº 6/2024-CGARB/DEDT/SVSA/MS: orientações para a vigilância da febre do Oropouche.

[8] Azevedo EAN, Silva AF, Silva VG, Machado LC, Lima GB, Ishigami BIM (2024). Genomic and phenotypic characterization of the Oropouche virus strain implicated in the 2022-24 large-scale outbreak in Brazil. J Med Virol.

[9] Ganjian N, Riviere-Cinnamond A (2020). Mayaro virus in Latin America and the Caribbean. Rev Panam Salud Pública.

[10] Hoch AL, Peterson NE, LeDuc JW, Pinheiro FP (1981). An outbreak of Mayaro virus disease in Belterra, Brazil. Am J Trop Med Hyg.

[11] Wiggins K, Eastmond B, Alto BW (2018). Transmission potential of Mayaro virus in Florida Aedes aegypti and Aedes albopictus mosquitoes. Med Vet Entomol.

[12] Coordenação-Geral de Vigilância de Arboviroses. Departamento de Doenças Transmissíveis. Secretaria de Vigilância em Saúde e Ambiente. Ministério da Saúde (2024). Orientações para a vigilância da febre do Oropouche.

[13] Pan American Health Organization (2024). PAHO urges countries to strengthen prevention, surveillance and diagnosis of the Oropouche virus following its geographic spread and recent clinical findings.

[14] Martins-Filho PR, Soares-Neto RF, Oliveira-Júnior JM, Santos CA (2024). The underdiagnosed threat of Oropouche fever amidst dengue epidemics in Brazil.. Lancet Reg Health Am.

[15] Martins-Filho PR, Carvalho TA, Santos CA (2024). Mayaro fever in Brazil from 2014 to 2024. J Travel Med.

[16] Forato J, Meira CA, Claro IM, Amorim MR, Souza GF, Muraro SP (2024). Molecular epidemiology of Mayaro virus among febrile patients, Roraima State, Brazil, 2018-2021. Emerg Infect Dis.

[17] Costa VG, Rezende Féres VC, Saivish MV, Lima Gimaque JB, Moreli ML (2017). Silent emergence of Mayaro and Oropouche viruses in humans in Central Brazil. Int J Infect Dis.

[18] Lima WG, Pereira RS, Cruz Nizer WS, Brito JCM, Godói IP, Cardoso VN (2021). Rate of exposure to Mayaro virus (MAYV) in Brazil between 1955 and 2018: a systematic review and meta-analysis.. Arch Virol.

[19] Lowe R, Lee S, Martins Lana R, Torres Codeço C, Castro MC, Pascual M (2020). Emerging arboviruses in the urbanized Amazon rainforest. BMJ.

[20] Celone M, Beeman S, Han BA, Potter AM, Pecor DB, Okech B (2024). Understanding transmission risk and predicting environmental suitability for Mayaro virus in Central and South America. PLOS Negl Trop Dis.

[21] Pereira-Silva JW, Ríos-Velásquez CM, Ribeiro G, Fabrício E, Matos C, Luiz S (2021). Distribution and diversity of mosquitoes and Oropouche-like virus infection rates in an Amazonian rural settlement. PLOS One.

[22] Delatorre E, Mendonça G, Gatti F, Có A, Pereira JP, Tavares E (2025). Emergence of Oropouche virus in Espírito Santo State, Brazil, 2024. Emerg Infect Dis.

[23] Vieira CJSP, Silva DJF, Barreto ES, Siqueira CEH, Colombo TE, Ozanic K (2015). Detection of Mayaro virus infections during a dengue outbreak in Mato Grosso, Brazil. Acta Trop.

[24] Nunes MRT, Souza WM, Savji N, Figueiredo ML, Cardoso JF, Silva SP (2019). Oropouche orthobunyavirus genetic characterization of full-length genomes and development of molecular methods to discriminate natural reassortments. Infect Genet Evol.

